# Procalcitonin: A Reliable Marker for the Diagnosis of Neonatal Sepsis

**Published:** 2012

**Authors:** Minoo Adib, Zahra Bakhshiani, Fakhri Navaei, Fereshteh Saheb Fosoul, Salomeh Fouladi, Hamidreza Kazemzadeh

**Affiliations:** 1 *Department of Immunology, School of Medicine, Isfahan University of Medical Sciences, Isfahan, Iran*; 2 *Department of Immunology, Fatema-Zahra Hospital, Isfahan of Social Security Organization (Treatment management), Isfahan, Iran *; 3 *Department of Pediatric, School of Medicine, Isfahan University of Medical Sciences, Isfahan, Iran*; 4 *Immunology Laboratory, Alzahra Hospital, Isfahan, Iran*

**Keywords:** C-reactive protein, Neonatal sepsis, Procalcitonin

## Abstract

**Objective(s):**

In the last few years, serum procalcitonin has been proposed as an early marker of infections in neonates, with varying results. In this study, we aimed to investigate the value of procalcitonin, and C- reactive protein in establishing the diagnosis of neonatal sepsis.

**Materials and Methods:**

Blood samples were collected at admission from 69 neonates with suspected infection (admitted to the Neonatal Intensive Care Units at Alzahra and Dr Beheshti Hospital in and Fatema-Zahra in Najafabad from May 2005 to April 2006). Patients were categorized in different groups according to clinical symptoms of sepsis, bacteriological and laboratory results. Group I consisted of 20 newborns with positive blood cultures and other biological tests which suggested infection. Group II consisted of 49 neonates with negative blood cultures but had two or three of clinical signs of sepsis. The control group included 18 healthy neonates with physiological hyperbilirubinemia and no clinical and biological data of infection, referred to the hospital for bilirubin determination. Procalcitonin and C-reactive protein (CRP) were determined by immunoluminometric assay and nephlometry method respectively.

**Results:**

Mean levels of procalcitonin and CRP in septic neonates (group I) were significantly higher than the other two groups (*P*< 0.005). Sensitivity, specificity, positive predictive value and negative predictive value were determined for all markers and compared with each other.

**Conclusion:**

We conclude that procalcitonin is a better marker than CRP in the diagnosis of neonatal sepsis.

## Introduction

Neonatal sepsis is a common cause of morbidity and mortality in newborn infants. Two patterns of disease, early- onset (<7 days of birth) and late-onset (>7 days) have been associated with neonatal sepsis (1, 2). Rapid diagnosis and treatment of systemic bacterial infection is essential in neonate and infants, since a delay in treatment of severe bacterial infection may not lead to a proper outcome (2, 3).

Clinical signs of systemic inflammation including changes in body temperature, tachycardia and routine laboratory tests like leukocytosis and C-reactive protein (CRP) are used for diagnosis of sepsis (4). Due to non-specific signs and symptoms of sepsis, the diagnosis of neonatal sepsis is quite difficult and can be misleading because critically ill neonates often manifest systemic inflammatory response syndrome (SIRS) without infection (3, 4). 

CRP is a good marker for diagnosis of neonatal sepsis. Elevated CRP levels are seen in infection, in autoimmune disease, in surgery, meconium aspiration and recent vaccination. Also, the CRP values do not rise significantly until almost 14-48 hr after the onset of infection (5-7).

Indeed, bacteriologic results need time and may be negative in newborns. Besides, it is impractical to obtain blood sample for serial blood culture from infants (5-7). So, new laboratory methods for early diagnosis of the diseases, evaluation of prognosis and treatment efficiency are needed.

Procalcitonin (PCT) has been proposed as a marker of bacterial sepsis in critically ill patients. PCT is a precursor of calcitonin and a 116 amino acids protein (8, 9).

 In contrast to calcitonin that has a short half-life of 10 min; PCT has a much longer half-life as 25-30 hr (10). 

In healthy persons, PCT levels are barely detectable (10). Although, the exact sites of production of PCT in sepsis have not been identified, monocytes and hepatic cells are believed to be potential sources (7, 11).

Bacterial lipopolysaccharide (LPS) has been shown to be a potent inducer of PCT release into the systemic circulation (12). Procalcitonin concentration starts to rise from 3-4 hr after an endotoxin challenge, peak about 6 hr, and remain increased for over 24 hr (7, 12). 

 In this study, we aimed to: investigate the value of PCT and CRP, in establishing the early diagnosis of neonatal sepsis.

## Materials and Methods

In a descriptive cross-sectional study, 69 at risk neonates who admitted to the Neonatal Intensive Care Units (NICU) at Alzahra and Dr Beheshti Hospital in Isfahan and Fatema-Zahra in Najafabad (from May 2005 to April 2006) were included. Written consent was obtained from the families of all the investigated neonates. 

The clinical criteria taken as indicative of sepsis were:

- Maternal risk factor such as fever, prolonged rupture of amniotic membrane >24 hr
- Neonatal history: low birth weight (< 2500 grams), premature birth (<37 weeks).
- Signs and symptoms of sepsis: feeding intolerance, lethargy, temperature instability, apnea, respiratory distress, poor perfusion, seizures, tachypnea, bradycardia, abdominal distension or vomits.

Neonates who had any features from I and II associated with two or more clinical symptoms of sepsis would warrant a septic screen (13).

Before initiation of antibiotic therapy in infants suspected of sepsis, blood samples for blood culture (1-2 ml), PCT and CRP measurements (1-2 ml) were obtained by peripheral venous puncture. 

 Serum was separated from blood cells by centrifugation and stored in 2 plastic tubes at -20 °C for measurements of PCT and CRP. The results of spinal fluid culture were obtained from the hospital laboratory.

Finally, according to clinical symptoms of sepsis, microbiologic and laboratory results, neonates classified in to three groups: 

1) Proven sepsis (n= 20): positive blood culture and clinical symptoms of sepsis.

 2) Suspected sepsis (N= 49): with clinical symptoms but negative blood culture. 

3) Control group (n= 18): healthy neonates with physiological hyperbilirubinemia (referred to the hospital for bilirubin determination) and no clinical and biological data of infection were selected as the control group. 

***Laboratory methods***

Serum C-reactive protein was determined by standard nephlometric method.

An immunoluminometric assay (LIAISON BRAHMS, ) was used for the specific measurement of PCT in serum (detection limit 0.10 ng/ml) following the instructions of the manufacturer. The assay uses two antigen-specific monoclonal antibodies that bind PCT (as an antigen) at different binding sites (the calcitonin and katacalcin segments). One of these antibodies was luminescence labeled (the tracer), and the other was coated on with magnetic particles (solid phase).

During the course of incubation, both antibodies react with PCT molecules in the sample to form “sandwich complexes”. As a result the luminescence labeled antibody is bound to the magnetic particles. 

Once the reaction is completed, the excess tracer is completely removed through washing.

Luminescence was measured automatically by an analyzer () and results were calculated using the software provided.

The Apgar score is used as an indicator of the infant’s condition in the first and fifth minutes after birth that include: appearance, heart rate, muscle tone, respiratory effort. Apgar score is measured by nurse-midwife or specialist.

***Microbiological examination***

One to two ml of blood was added to blood culture media (Biphasic) and incubated at 37 °C for 5-7 days (22, 23). Bottles with positive results were sub cultured on blood agar () and EMB media. The isolated microbes were identified by standard bacteriological methods.

***Statistical analysis***

To compare means of the variables, one-way ANOVA test was done by SPSS (version 11.5). Categorical variables between groups were analyzed using Chi-square test.

Diagnostic efficiency was defined as sensitivity, specificity, positive predictive value (PPV) and negative predictive value (NPV). Results were presented as percent (%), mean, standard deviation (SD), median, and minimum-maximum (min-max). A *P-*value< 0.05 was considered as significant.

## Results

In this study, 20 neonates with positive blood cultures and clinical sepsis (group I or patient group) and 49 suspected sepsis neonates (group II) and 18 healthy neonates (group III) were enrolled. Patient group was evaluated in two subgroups as early-onset (n= 13) and late onset neonatal sepsis (n= 7).

Blood cultures were positive for all patients (group I). The identified bacteria included *Staphylococcus aureus* (n= 6), coagulase negative *Staphylococcus *(n= 6), *Streptococcus *beta hemolytic group A (n= 3), *Escherichia coli* (n= 3), *Pseudomonas aeruginosa* (n= 1) and *Entrobacter* (n= 1).

The demographic data of the proven, suspected sepsis and control groups are shown in Table 1. A statistical significant difference was observed between the mean of birth weight in septic and suspected groups in comparison with control group (*P*< 0.05). The mean of gestational age (GA) in proved sepsis infants was 31.9 weeks that was lower than two other groups (*P*< 0.05).

The mean of CRP and PCT in studied groups are shown in Table 2. There was a significant difference between the mean of CRP level in healthy controls and septic infants (*P*< 0.05). In addition, it was observed a significant difference between septic and suspected newborns (*P*< 0.05).

**Table 1:** The characteristics of studied groups include the mean of birth weight, gestational age (GA), Apgar score

Features	Proved sepsis	Suspected sepsis	Control	*P *-value
Nu. of neonates	20	49	18	
Birth weight (g)	2098±621	2313±826	2756±437	0.002
GA (week)	31.94±4.2	33.9 ±3.5	36.5±8.4	0.04
Apgar scor1	6.29±2.3	6.70±2.75	8.1±1.30	0.189
Apgar scor5*	7.8±1.57	8.4±1.84	9.3±0.84	0.157

*The Apgar score is used as an indicator of the infant’s condition in the first and fifth minutes after birth that include: appearance, heart rate, muscle tone, respiratory effort.

**Table 2:** The mean and standard deviation of CRP and PCT in different groups

Markers		Mean±SD	*P*- value
CRP mg/l	Control group	4.22 ± 2.66	*P*< 0.05
	Proved sepsis	23.16±32.6	*P*< 0.05
	Suspected sepsis	9.31±13.60	*P*< 0.05
PCT ng/ml	Proved sepsis	5.70±8.72	*P*< 0.05
	Control group	0.69±0.54	P< 0.05
	Suspected sepsis	3.03±5.94	P< 0.05

**Figure 1:**
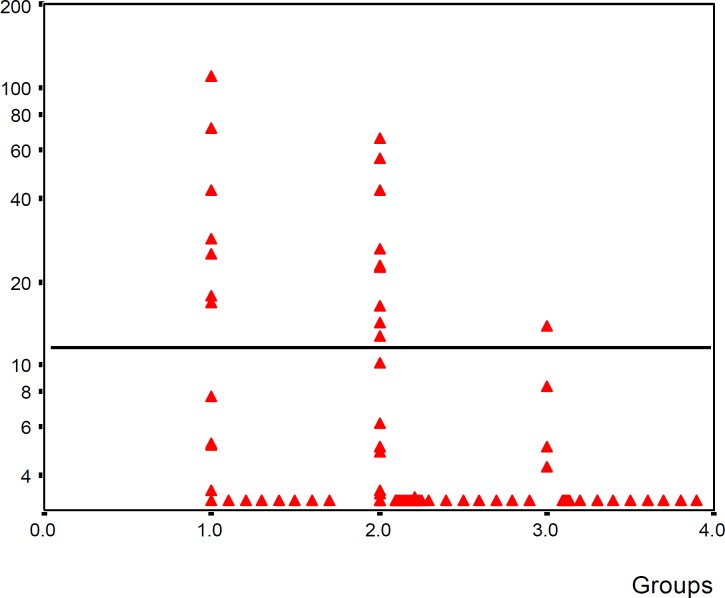
Comparison of CRP concentration in three groups (group I: proved sepsis, group II: suspected sepsis, group III: control). Horizontal line shows cut-off value = 12 mg/l

CRP concentration in 35% of proved sepsis group was higher than the cut-off value. But in suspected sepsis 17% of cases and in the control group only in 5% of infants, CRP level was located higher than the cut-off value (Figure 1).

CRP level in 33.3% (4 of 12 neonate) of early onset sepsis and 57% (4 out 7 cases) of late onset sepsis were upper than cut-off value. 

**Figure 2:**
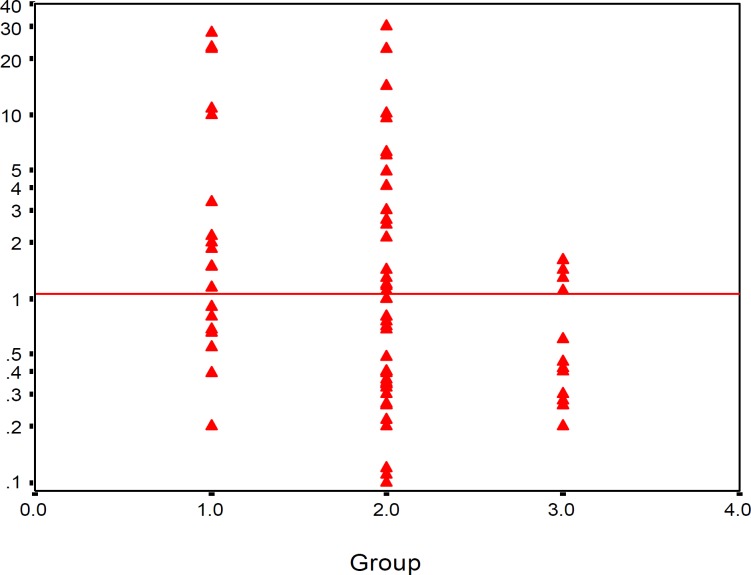
Comparison of PCT concentration in three groups (group I: proved sepsis, group II: suspected sepsis, group III: control). Horizontal line shows cut-off value = 1.1 ng/ml.

PCT level was significantly higher in septic and suspected infants in comparison with the normal infants (*P*< 0.05). PCT level in 30% of proved sepsis group and 40% of suspected sepsis was located lower than the cut-off value, but in 77.8% of infants in the control group it was located lower than the cut-off value (Figure 2). The optimum cut-off value was found to be 12 mg/l for CRP and 1.1 ng/ml for PCT.

At a cut-off value, 12 mg/l CRP was found to have a sensitivity of 45%, specificity of 95%, positive predictive value (PPV) of 30%, negative predictive value (NPV) of 30% for the diagnosis of neonatal sepsis. 

We found 70% sensitivity, 80% specificity, 80% PPV and 75% NPV for procalcitonin as a marker for the early diagnosis of neonatal sepsis.

*some points are over labs

## Discussion

In recent years measurement of procalcitonin and other inflammatory mediators have been reported as sensitive parameters for the early diagnosis of neonatal sepsis and evaluating its outcome (10, 13). Varieties of proinflammatory cytokines plays a role in pathogenesis of bacterial sepsis. Production of interleukin-6 occurs before procalcitonin. These cytokines seems to trigger the procalcitonin secretion from target cells (14, 15).

In some studies with cut-off value of 10 mg/l for CRP, the range of reported statistical outcomes is as follows: sensitivity 70% to 93%; specificity 41% to 98%; positive predictive accuracy 6% to 83%; and negative predictive accuracy 97% to 99% (16).

In our study CRP: 12 mg/l was found to be the most appropriate cut-off value by using receiver operating characteristic (ROC) curves, and at this cut-off value, test sensitivity was 45%, specificity was 95%, PPV was 30% and NPV was 30%.

The increase in the serum concentration of CRP is rather slow during the first 24-48 hr of infection and this may negatively affect the sensitivity of the test. In addition, increase in CRP concentration in non-infected clinical conditions such as meconium aspiration, prolong rupture of membranes are thought to affect the specificity of the test (17).

PCT has been intensively investigated for its diagnostic role in neonatal sepsis. It has been reported that high concentration of plasma PCT was found in infants with severe infection, while PCT levels were very low in those with no infections (8). Many authors found that procalcitonin is a promising marker for the diagnosis of neonatal sepsis (10, 12, 18). In these studies, PCT sensitivity in the early diagnosis of neonatal sepsis was found to be 83-100% while the specificity was 70-100% (9, 12, 18). But some investigators questioned the diagnostic accuracy of PCT in detecting of neonatal sepsis. In these studies, it was reported that serum levels had also increased in non-infected neonates with perinatal asphyxia, intracranial hemorrhage, pneumothorax, or after resuscitation, and these conditions had negatively affected the specificity of PCT (19, 20). In the research of Chin yi-ling *et al* (2004) sensitivity of 69.5% and specificity of 64.5% for PCT were obtained, compared to 67.25% of sensitivity and 93.9% of specificity for CRP (21). Janota *et al* (2001) indicated the sensitivity and specificity of 75% and 59% for procalcitonin respectively (20). In their study, Chiesa *et al* (1998) reported that a serum PCT rise caused by prenatal events other than infection was smaller than the PCT response against infection (8).

We found 75% sensitivity, 80% specificity 80%, positive predictive value and 75% negative predictive value for procalcitonin as a marker for the early diagnosis of neonatal sepsis. Also, the results of study by Khoshdel *et al* (2008) in Iran (Shahrekord) showed that sensitivity, specificity, and positive and negative predictive values of PCT level for neonatal sepsis were 87.5%, 87.4%, 30.4%, and 99.1% respectively (23). The study of Zahedpasha *et al* (2009) in showed that PCT levels were remarkably high in neonates with proven sepsis and the levels dropped dramatically after treatment with antibiotics (24).

In the present study, among 20 neonates of proved sepsis, 6 cases had PCT levels less than 1.1 ng/ml (cut-off value). In the control group, among 18 neonates, 4 neonates had procalcitonin higher than 1.1 ng/ml, perhaps due to physiological increase of procalcitonin, reported up to 21-48 hr postpartum, even in the absence of infection. Kocabas (2007) concluded that PCT and tumor necrosis factor-α are best markers in the diagnosis of neonatal sepsis in comparison with IL-6, IL-8 and CRP (17).

## Conclusion

Our results indicated that the sensitivity of procalcitonin (70%) was higher than CRP (45%) for the diagnosis of neonatal sepsis and PCT appears to be a useful marker for the severity of infection. 
